# Lower systemic inflammation is associated with gut firmicutes dominance and reduced liver injury in a novel ambulatory model of parenteral nutrition

**DOI:** 10.1080/07853890.2022.2081871

**Published:** 2022-06-15

**Authors:** Ashish Samaddar, Johan van Nispen, Austin Armstrong, Eric Song, Marcus Voigt, Vidul Murali, Joseph Krebs, Chandra Manithody, Christine Denton, Aaron C. Ericsson, Ajay Kumar Jain

**Affiliations:** a Department of Pediatrics, Saint Louis University School of Medicine, St. Louis, MO, USA; b Department of Veterinary Pathobiology, College of Veterinary Medicine, University of Missouri, Columbia, MO, USA; c Department of Pharmacology and Physiology, Saint Louis University School of Medicine, St. Louis, MO, USA

**Keywords:** Microbiota, *Firmicutes*, *Ruminococcaceae*, lipopolysaccharide, inflammation, total parenteral nutrition

## Abstract

**Background:**

Total Parenteral Nutrition (TPN) provides lifesaving nutritional support to patients unable to maintain regular enteral nutrition (EN). Unfortunately, cholestasis is a significant side effect affecting 20–40% of paediatric patients. While the aetiology of TPN-associated injury remains ill-defined, an altered enterohepatic circulation in the absence of gut luminal nutrient content during TPN results in major gut microbial clonal shifts, resulting in metabolic endotoxemia and systemic inflammation driving liver injury and cholestasis.

**Hypothesis:**

To interrogate the role of gut microbiota, using our novel ambulatory TPN piglet model, we hypothesized that clonal reduction of bacteria in *Firmicutes* phylum (predominant in EN) and an increase in pathogenic Gram-negative bacteria during TPN correlates with an increase in serum lipopolysaccharide and systemic inflammatory cytokines, driving liver injury.

**Methods:**

Upon institutional approval, 16 animals were allocated to receive either TPN (*n* = 7) or EN only (*n* = 9). The TPN group was subdivided into a low systemic inflammation (TPN-LSI) and high systemic inflammation (TPN-HSI) based on the level of serum lipopolysaccharide. Culture-independent identification of faecal bacterial populations was determined by 16S rRNA.

**Results:**

Piglets on TPN, in the TPN-HSI group, noted a loss of enterocyte protective Firmicutes bacteria and clonal proliferation of potent inflammatory and lipopolysaccharide containing pathogens: *Fusobacterium*, *Bacteroidetes* and *Campylobacter* compared to EN animals. Within the TPN group, the proportion of *Firmicutes* phylum correlated with lower portal lipopolysaccharide levels (*r* = −0.89). The TPN-LSI had a significantly lower level of serum bile acids compared to the TPN-HSI group (7.3 *vs.* 60.4 mg/dL; *p* = .018), increased day 14 weight (5.67 *vs.* 5.07 kg; *p* = .017) as well as a 13.7-fold decrease in serum conjugated bilirubin.

**Conclusion:**

We demonstrate a novel relationship between the gut microbiota and systemic inflammation in a TPN animal model. Pertinently, the degree of gut dysbiosis correlated with the severity of systemic inflammation. This study underscores the role of gut microbiota in driving liver injury mechanisms during TPN and supports a paradigm change in therapeutic targeting of the gut microbiota to mitigate TPN-related injury.

**KEY MESSAGES**
- This study identified a differential link between gut microbiota and inflammation—the higher the dysbiosis, the worse the systemic inflammatory markers.
- Higher levels of *Firmicutes* species correlated with reduced inflammation.

## 1. Introduction

Total Parenteral Nutrition (TPN) is the process of providing all nutritional needs intravenously. It remains an essential lifesaving therapy in individuals who cannot tolerate enteral nutrition (EN). While the benefits of this widely popular worldwide therapy remain undisputed, enthusiasm is tempered due to a significant association of adverse effects. TPN-associated liver disease is a well-defined progressive cholestatic liver injury occurring in patients on TPN.

While many prior studies have focussed on possible detrimental effects from the constituents of the TPN solution including lipids [1,2], emerging data support [3,4] that the state of luminal content deprivation during TPN induces alterations in gut-systemic signals, which drives liver injury mechanisms.

Indeed, due to a lack of luminal nutrition, there are significant alterations in the gut microbiota as well as gut signalling in animals on TPN [5–8]. Emerging data demonstrate the dominance of the *Firmicutes* phylum in normal enteral nutrition (EN)-fed animals, whereas TPN results in major gut microbial clonal shifts, resulting in metabolic endotoxemia [5].

Literature supports that pathogenic biofilm-forming and pro-inflammatory bacterial genera like *Bacteroides*, *Fusobacterium*, and *Campylobacter* promote intestinal inflammation [9,10], increase intestinal permeability [11,12], and enable bacterial flux across the injured gut mucosa resulting in cytokine-mediated hepatocellular injury [13–15].

Given that TPN is known to variably drive liver injury [4,6,16], with some individuals being protected from its side effects, we hypothesized that the degree of systemic inflammatory injury would correspond to the relative abundance of phyla *Firmicutes* and *Bacteroidetes*, and sub-phylum taxa, such as *Fusobacterium* and *Campylobacter*.

To test our hypothesis, we used our published, large animal TPN piglet model. The rationale for using this model system underscores the fact that rodents and other small animal models do not adequately recapitulate the human TPN-dependent condition [17,18]. Complex surgical procedures like placement of indwelling catheters for TPN and enteral nutrition support are more translational to the human TPN delivery in a larger animal like a piglet [17–19]. Additionally, the extensive homology of the porcine liver and gastrointestinal tract in both form and function in humans presents great advantages to the piglet model [20–23].

Thus, addressing shortcomings of prevailing model systems, we have established a novel untethered advanced ambulatory TPN piglet model using miniature pumps, jugular and duodenal catheters (SLU#2346,43-R-011) to closely recapitulate human TPN delivery [17].

Using this system, we prospectively evaluated the taxonomic and functional composition of the gut microbiota in piglets on EN and those receiving TPN. Furthermore, we set forth to understand whether there was a relationship between the degree of gut dysbiosis or preponderance of specific taxa and the severity of systemic inflammation.

## 2. Methods

### 2.1. Animal procurement

Saint Louis University (SLU) is a registered research facility recognized by the United States Department of Agriculture (USDA). The study was approved by and conducted in accordance with the Institutional Animal Care and Use Committee (IACUC) of SLU (SLU No. 2657, US Department of Agriculture registration 43-R 011) as well as the Guide for the Care and Use of Laboratory Animals (Garber, Barbee). The neonatal piglets used for the study were seven to ten days old and procured from a class A vendor approved by the university.

### 2.2. Acclimatization and housing

Neonatal piglets were placed in heated cages for three days, from the time of arrival to the start of the study. Throughout the study, an SLU facility veterinarian monitored animal health and well-being.

### 2.3. Surgery and catheter placement

After acclimatizing for three days, catheters were surgically placed in the piglets as published [8]. Three to five percent isoflurane was used for anaesthesia induction in the veterinary operating room. Each animal was then transferred for surgery. The anaesthetic agent isoflurane (two to four percent) was administered *via* a cone mask intraoperatively. Vital signs, oxygen saturation, heart rate, respiratory rate, and temperature were continuously monitored. Subsequently, the abdomen and neck were draped and made ready for aseptic surgery.

#### 2.3.1. Bilateral jugular catheter placement

As published [8], through a simple vascular cut-down technique, two internal jugular vein catheters were positioned. Vessels and the catheters are securely connected. Sterile heparinized saline (3 mL, 3 u/mL) was injected to confirm patency. To exit the skin, the catheter was tunnelled subcutaneously caudal to the scapulae.

#### 2.3.2. Duodenal catheterization

A 5 cm midline abdominal incision was created. The duodenum was exposed by excising a portion of the non-glandular stomach. A 20-gauge hypodermic needle was used to make a full thickness poke incision followed by the placement of a sterile silastic catheter. The catheter was secured to the duodenum with non-absorbable sutures through the use of a purse string pattern. To further secure and prevent unintentional leakage of intestinal content into the peritoneal cavity, the serosa of the duodenum was folded around the catheter. The catheter and abdominal wall were sutured together.

The catheters were exteriorized through the same incision caudal to the scapulae. The catheters were flange secured *via* a purse string suture at the body wall. A single layer of an absorbable suture was used to close the body wall. The skin was opposed in a continuous subcuticular pattern with absorbable sutures and closed by surgical staples. Surgical staples and two interrupted non-absorbable monofilament sutures and surgical staples were used to secure all catheters.

#### 2.3.3. Jacket placement

Animals were fitted with a custom pre-conditioned/ambulatory jacket that had pockets on both sides. An ambulatory battery-operated infusion pump (OrchesTA 500, PA, USA) was placed in the right pocket, and tubing was placed in the left pocket. The duodenal catheter was sieved through the right pocket and saline locked. The jugular catheter was sieved through the left pocket fabric and connected to the pre-conditioned pump.

### 2.4. Recovery

The piglets were closely monitored until fully recovered. Once recovered, the animals were returned to their home cages. Subsequently, as published [8,22], enteral nutrition or TPN was provided in EVA bags (Medtec Medical, EVA, Product code 66050) placed in the left pocket and connected to respective catheters.

### 2.5. Animal monitoring

An SLU veterinarian and the study team examined and monitored the animals over the course of the study. Each morning, research personnel weighed the piglets and changed the nutrition bags. All visits were in accordance with the IACUC at SLU and the Guide for the Care and Use of Laboratory Animals.

### 2.6. Nutrition intake

Throughout the experimental period, isocaloric nutrition was provided to all animals. As published [3,8,22], the enteral nutrition (EN) group received the swine replacement formula LitterLife, Merrick’s Inc., WI, USA *via* the duodenal catheter. TPN was delivered using the commercially available Parenteral Nutrition preparation (Clinimix E, Baxter, IL, USA) and Intralipid (Fresenius Kabi, Germany) *via* the jugular catheter.

### 2.7. Group allocation

Sixteen animals were randomly allocated into one of two different groups post-surgery recovery. In the EN group (*n* = 9), animals were provided enteral nutrition and no TPN. The TPN group received TPN (*n* = 7) and no EN.

We have previously published significant gut microbial shifts in animals on TPN [3,5,6], however, these microbial clonal changes and the accompanying liver injury presented large variability, calling into question if specific clonal shifts would drive higher serum LPS and liver injury.

Leveraging this concept, the TPN group was further subdivided into two groups, low systemic inflammation (TPN-LSI) and high systemic inflammation (TPN-HSI) based on a portal serum lipopolysaccharide discriminatory level of 22 ng/mL. While there are no established cut-offs, with limited literature on porcine TPN models for this novel study, LPS, the threshold was generated by using the median LPS value across the TPN animals. Similarly, as a cytokine correlates, serum IL-6 at 1.7 pg/mL cut-off was also utilized to dichotomize the TPN groups to provide additional rigour, using a similar methodology.

### 2.8. Animal euthanasia and sample collection

At the end of the study, after 14 days, piglets were euthanized, as published, in accordance with the American Veterinary Medical Association (AVMA) Guidelines using intravenous injection of entobarbital sodium (100 mg/kg) [6,8,17]. As described previously, the abdomen was opened post-euthanasia and the entire small intestine distal to the stomach up to the ileocecal junction was removed. The small intestine was immediately flushed with cold saline, its contents extruded, and the tissue weighed. Portal and peripheral blood were collected. The cytokine analysis was performed using a multiplex cytokine assay kit (Millipore Cat # HSTCMAG-28sk). Portal LPS levels were measured *via* a porcine LPS ELISA Kit (MyBioSource Catalog # MBS2513363). Serum samples were analyzed by an automated analyzer at the SLUCare facility as described previously [3,17,24].

### 2.9. Stool collection

Stool samples were collected at the start of the study (Day 1) and before animal euthanasia (Day 14). These fresh samples were later stored in 2.0-mL sample processing beaded tubes (S6003-50; Zymo Research, Irvine, CA, USA) and placed in a 4 °C refrigerator.

### 2.10. DNA extraction

DNA extractions were carried out using the Xpedition Soil/Faecal DNA Miniprep (D6202 was used to carry out the DNA extractions; Zymo Research). Within 8 h of procurement, a lysis and storage buffer (1.5 mL) that came with the kit was added to each 2.0-mL tube and then processed with the Xpedition Sample Processor (Zymo Research). NanoDrop ND2000 Spectrophotometer (Thermo Scientific, Waltham, MA, USA) was used to assess endpoint DNA quality and quantity. Final DNA yields were quantified through fluorometry (Qubit 2.0, Invitrogen, Carlsbad, CA, USA) using quant-iT BR dsDNA reagent kits (Invitrogen).

### 2.11. 16 s rRNA library preparation and sequencing

Stool DNA was later handled at the University of Missouri DNA Core Facility. The V4 portion of the 16S rRNA gene coupled with universal primers (U515F/806R) was used to construct each bacterial 16S rRNA amplicon through an application process. To identify the V4 region, a standard adapter sequence by Illumina was used. proBase was used to obtain the Oligonucleotide sequences. Dual indexed forward and reverse primers were used in all reactions. Polymerase Chain Reaction was undergone in 50 µL reactions containing 100 ng metagenomic DNA, dNTPs (200 µM each), primers (0.2 µM each), and Phusion high-fidelity DNA polymerase (1 U). The specific application sequence was 98 °C (3:00) + [98 °C (0:15) + 50 °C (0:30) + 72 °C (0:30)] × 25 cycles + 72 °C (7:00). Axygen Axyprep MagPCR clean-up beads were then added to the processed and purified amplicon pools (5 µL/reaction) to 50 µL of amplicons and incubated at room temperature for 15 min. The resulting products were rinsed in 80 percent ethanol. Afterwards, the dried pellet was resuspended in 32.5 µL EB buffer and incubated at room temperature for 2 min, and then for 5 min, the specimen was kept on the magnetic stand. A Fragment Analyzer automated electrophoresis system by Advanced Analytical was used to evaluate the final amplicon pool. The sample was later quantified using quant-iT HS dsDNA reagent kits and diluted on the MiSeq instrument as per the Illumina standard for sequencing.

### 2.12. Informatics analysis

The University of Missouri Informatics Research Core Facility performed the reading, merging, clustering, and annotation of DNA sequences. FLASH software was used to merge paired DNA sequences. Cutadapt (https://github.com/marcelm/cutadapt) excised primers at both ends of the contig as well as cull contigs not containing both primers. The usearch fastq_filter command (http://drive5.com/usearch/manual/cmd_fastq_filter.html) allowed for convenient modification of contigs, rejecting those where the expected number of errors exceeded 0.5. Contigs were all shortened to 248 bases with smaller contigs were removed. Qiime 1.9 function split_libraries_fastq.py was then utilized to demultiplex the contigs. Sample outputs were then clustered using a single combined file. The uparse command (http://www.drive5.com/uparse/) allowed for both clustering contigs with 97% identity and removing chimaeras. Taxonomy was given to particular OTUs by using BLAST on the SILVA database v13210 of 16S rRNA taxonomy and sequences.

### 2.13. Data analysis

Statistical Analysis: Graph Pad Prism version 7.03 software was utilized for statistical analysis. Descriptive statistics on outcomes were calculated as the median and interquartile range (IQR). Pairwise Mann–Whitney *U* tests were conducted for assessing serological markers and histology reads. All tests were two-sided and used a significance level of 0.05.

Principal Coordinate Analysis (PCoA) and permutational multivariate analysis of variance (PERMANOVA) was used to determine across-group gut microbiota differences in beta-diversity. A nonparametric *t*-test was used to identify the significant clades at an alpha level of 0.05. Serum conjugated bilirubin and bile acids were used as a surrogate for hepatic cholestasis.

## 3. Results

### 3.1. Weight gain

**Figure 1.**
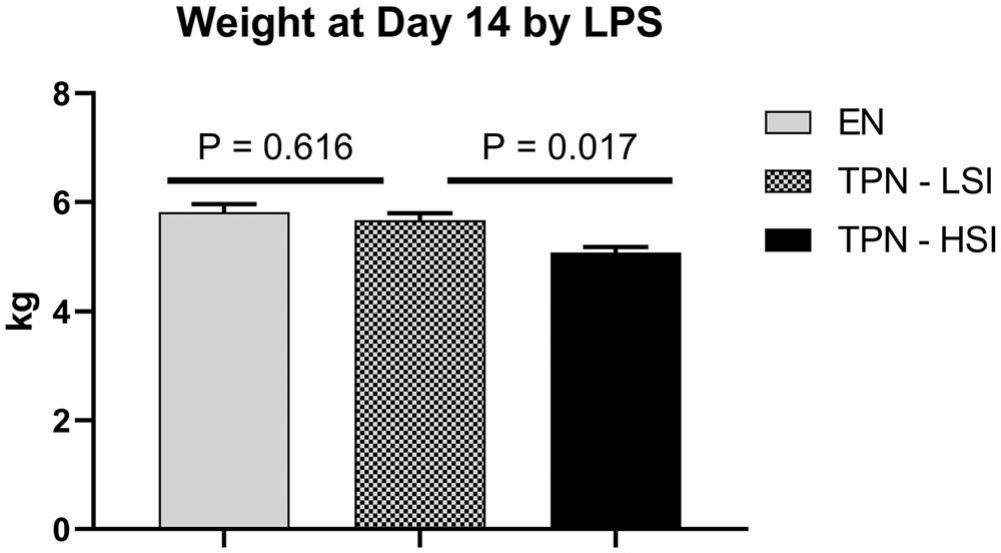
Day 14 weight. A significant decrease in day 14 weight was seen in the TPN-HSI pigs compared to TPN-LSI. No significant change in weight was seen in TPN-LSI compared to pigs fed enteral nutrition. Pairwise *t*-tests were performed to determine the *p*-value. All tests were two-sided using a significance level of 0.05.

A total of 16 piglets were included in the analysis. To characterize the impact of the inflammatory state on weight gain we compared end-of-study weights between the groups. As shown in Figure 1, the TPN-LSI group had no significant difference in weight gain *vs.* EN animals (*p* = .616). However, there was a statistically significant lower weight gain in TPN-HSI animals *vs.* TPN-LSI (*p* = .017).

### 3.2. Gut microbiota

**Figure 2.**
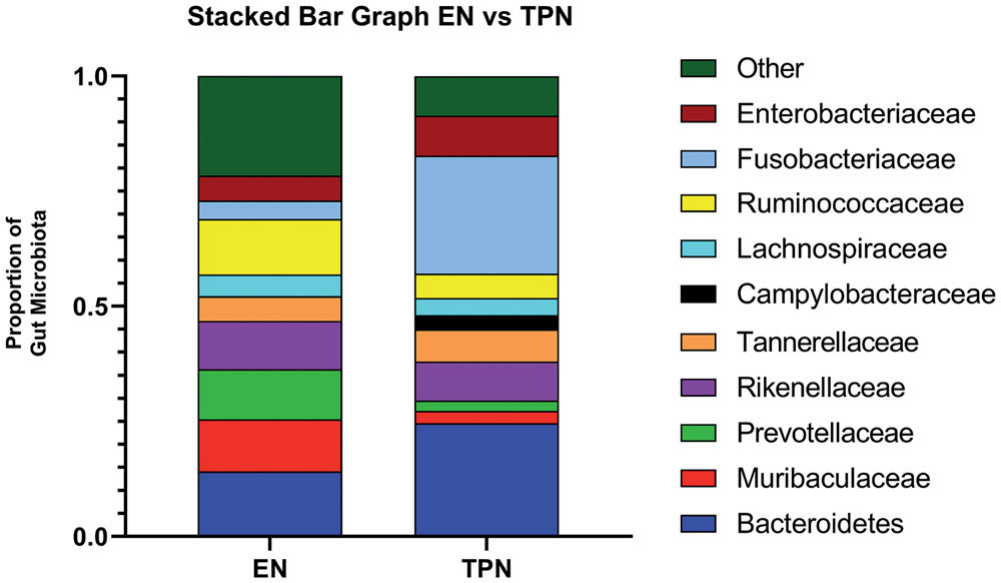
Gut microbiota. The TPN group had significantly decreased proportions of Ruminocococceae (5.3 *vs.* 12.0%; *p* = .016) and Muribaculaceae (2.7 *vs.* 11.3%; *p* = .042) compared to piglets fed milk *via* enteral nutrition. TPN piglets had significantly increased proportions of Fusobacteria (25.6 *vs.* 4.0%; *p* = .001) and Epsilonbacteria (3.1 *vs.* 0.1%; *p* = .005) compared to piglets fed enterally. Gut microbiota classified by 16S rRNA in stool samples. Pairwise Mann–Whitney *U* test were performed to determine the *p*-value. All tests were two-sided using a significance level of 0.05.

Bacterial sequences were classified with the RDP Classifier algorithm. TPN had significant microbial shifts compared to EN animals (Figure 2). Specifically, the TPN group had a significantly decreased proportion of *Ruminococcaceae* (5.3 *vs.* 12.0%; *p* = .016) and *Muribaculaceae* (2.7 *vs.* 11.3%; *p* = .042) and significantly increased proportions of Fusobacteria (25.6 *vs.* 4.0%; *p* = .001) as well as *Campylobacteraceae* (3.1 *vs.* 0.1%; *p* = .005).

### 3.3. Gut microbiota changes and inflammation

**Figure 3.**
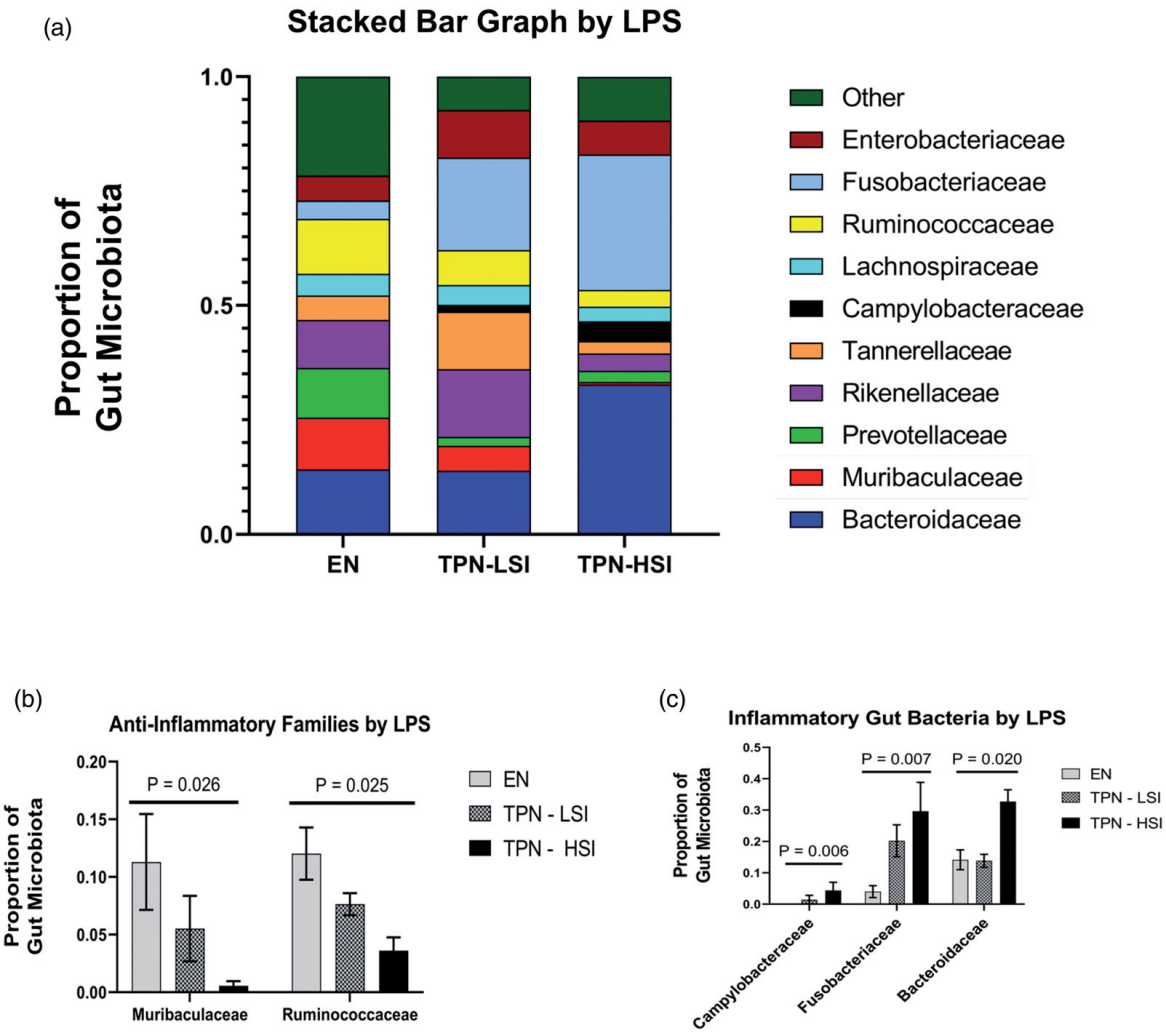
(A) Gut microbiota by portal serum LPS level. (B) Key inflammatory bacteria by LPS level. TPN-HSI had a 3.02, 1.47, and a 2.36-fold increase in the proportion of inflammatory gram-negative Campylobacteraceae, Fusobacteriaceae, and Bacteroidaceae families compared to the TPN-LSI group, respectively. (C) Key anti-inflammatory bacteria by LPS level. TPN-HSI group had a 9.86- and 2.11-fold reduction in the level of beneficial Muribaculaceae and Ruminococceae, respectively compared to that of the TPN-LSI group. (A–C) Gut microbiota classified by 16S rRNA in stool samples. (B,C) Kruskal–Wallis *H* tests were performed to determine the *p*-value. All tests were two-sided using a significance level of 0.05.

Within, the TPN group, the degree of gut dysbiosis had strong correlations to the severity of systemic inflammation (Figure 3(a–c)). TPN-HSI group had a 3.02-, 1.47-, and a 2.36-fold increase in the proportion of Gram-negative families *Campylobacteraceae*, *Fusobacteriaceae*, and *Bacteroidaceae* compared to TPN-LSI animals (Figure 3(a,b)). Additionally, the TPN-HSI animals had a 9.86- and 2.11-fold reduction in the level of *Muribaculaceae* and *Ruminococcaceae*, respectively compared to the TPN-LSI group. These families are known to produce short-chain fatty acid (SCFA), propionate, and butyrate, needed by enterocytes (Figure 3(a,c)).

**Figure 4.**
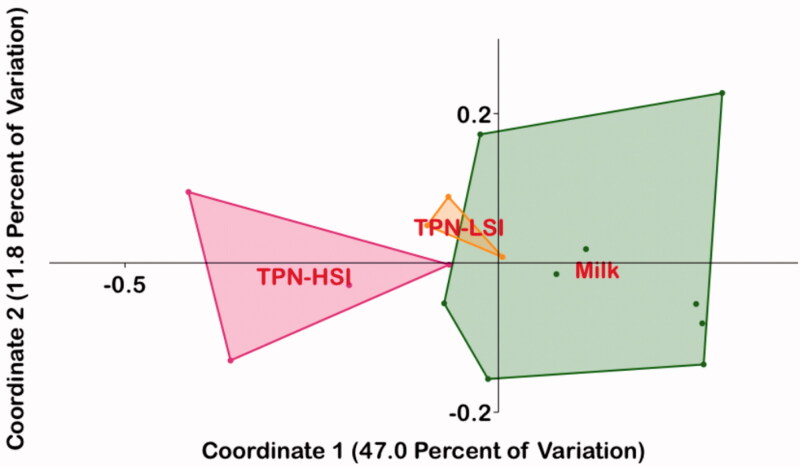
Principal coordinate analysis (PCoA). The microbiota composition of TPN-LSI had more overlap with that of the EN group compared to the gut microbiota profile of the TPN-HSI group. Bray-Curtis coordinates were used. Gut microbiota classified by 16S rRNA in stool samples. Through PERMANOVA analysis, the overall composition of gut microbiota in the enteral animals was statistically significant from that of the high inflammation TPN group (Bonferroni-corrected *p*-value = .0075). The Bonferroni-corrected *p*-value between the gut microbiota composition of the enteral animals and the lower inflammation was not significant at a value of 0.115.

We next performed the Principal Coordinate Analysis (PCoA) of 16S family level data using Bray-Curtis similarities. The microbiota composition of TPN-LSI animals showed a significant overlap with that of the EN animals, with non-overlapping profiles for the TPN-HSI group (Figure 4). Using PERMANOVA analysis, the overall composition of gut microbiota in EN was significantly different from the TPN-HSI group (Bonferroni-corrected *p* = .0075) with no significant difference *vs.* TPN-LSI animals (Bonferroni-corrected *p* = .115).

### 3.4. Cytokine analysis

The inflammatory cytokines had a strong correlation with LPS in both the TPN and EN groups. For the TPN group they were: IL-6 (*r* = 0.996) and IL-1β (*r* = 0.968) (Figure S1). For the EN group these correlations were: IL-6 (*r* = 0.979) and IL-1β (*r* = 0.974) (Figure S2).

**Figure 5.**
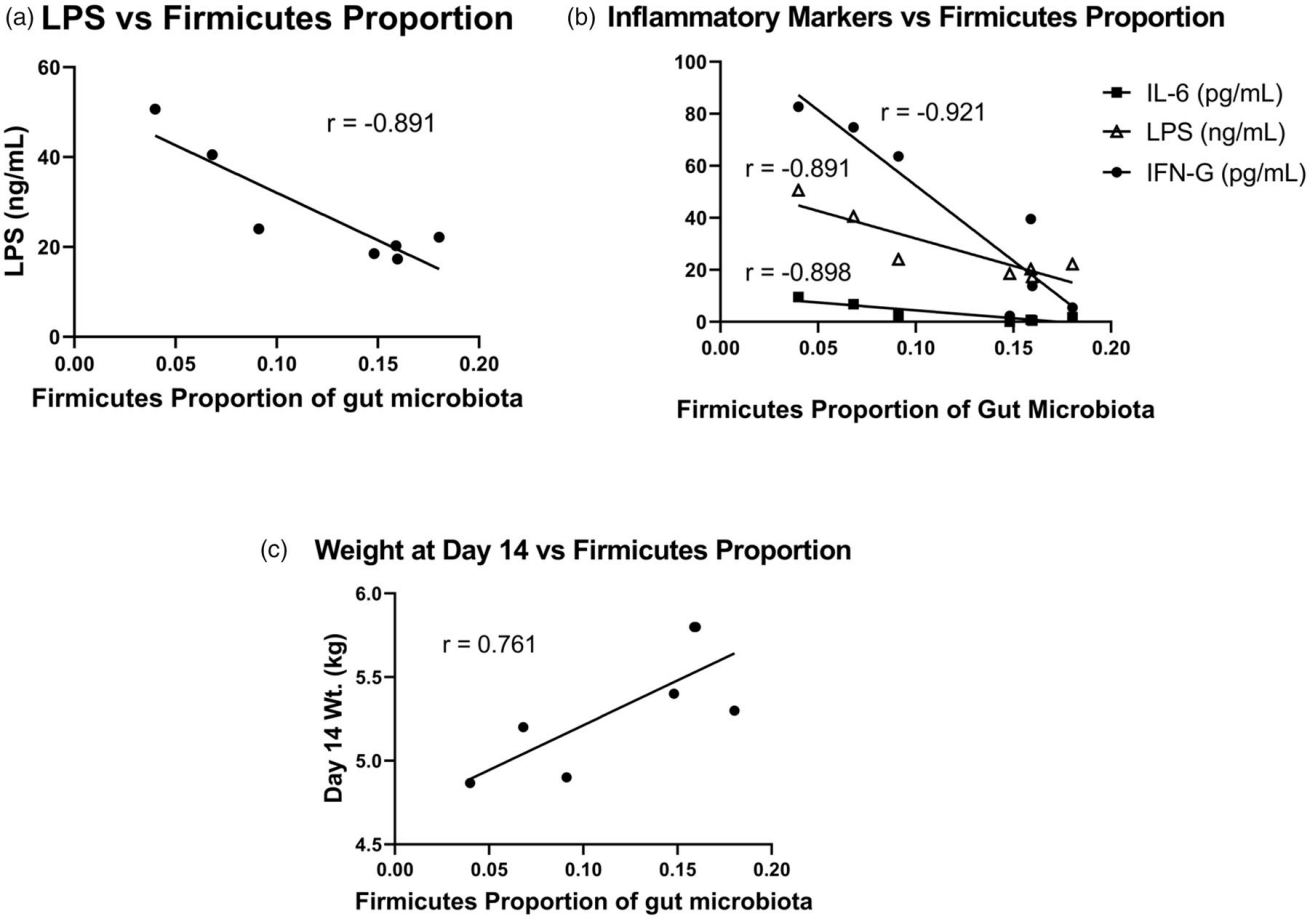
(A) LPS levels *vs.* Firmicutes proportion. (B) Inflammatory cytokine levels *vs.* Firmicutes proportion. In the TPN group, the proportion of gut firmicutes had a strong negative correlation with the pro-inflammatory cytokines: IL-6 (*r* = −0.898), IFN-G (*r* = −0.921), and LPS (*r* = −0.891). (C) Day 14 Weight *vs.* Firmicutes. Within the TPN group, the proportion of Firmicutes had a strong positive correlation to the day 14 weight (*r* = 0.761). Inflammatory cytokines that had the strongest negative correlation with day 14 weight were: IL-6 (*r* = −0.695) and IL-1B (*r* = −0.735).

The proportion of gut *Firmicutes* had a strong negative correlation with the pro-inflammatory cytokines: IL-6 (*r* = −0.898), IFN-g (*r* = −0.921), and LPS (*r* = −0.891) in TPN animals (Figure 5(a,b)).

In both the TPN and EN groups, inflammatory mediators were negatively correlated with the day 14 weight. Within the TPN group, the proportion of *Firmicutes* had a strong positive correlation to the day 14 weight (*r* = 0.761) (Figure 5(c)).

In animals on TPN, the inflammatory cytokines with the strongest negative correlation were: IL-6 (*r* = −0.695) and IL-1β (*r* = −0.735) (Figure S3). Meanwhile, in the EN group, the mediators with the strongest correlation to the final weight were IL-1β (*r* = −0.631) and IFN-g (*r* = −0.711) (Figure S4).

### 3.5. Markers for PNALD

**Figure 6.**
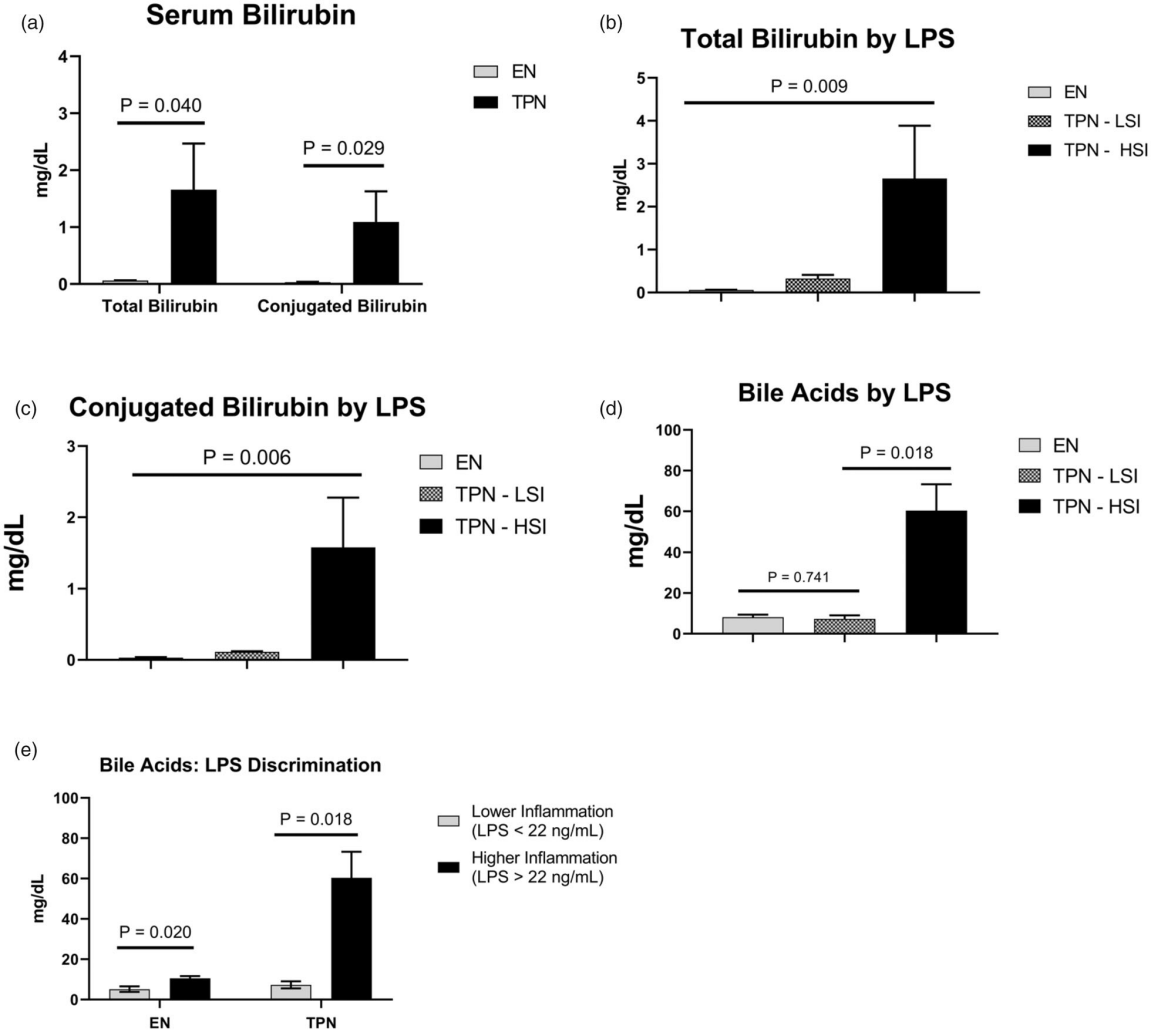
(A–C) Serum bilirubin. TPN-HSI animals had a 4.84- and 13.7-fold increase in the total and conjugated bilirubin level, respectively compared to the TPN-LSI group. (D–E) Bile acid level. TPN-HSI had a significant increase in serum bile acid levels compared to TPN-LSI. Pigs fed enterally had a higher significant increase in bile acids depending on systemic inflammation levels. (A,D,E) Pairwise *t*-tests were performed to determine the *p*-value. (B,C) ANOVA test was performed to determine the *p*-values. All tests were two-sided using a significance level of 0.05.

Serum conjugated bilirubin is a well-characterized surrogate for hepatic cholestasis. We assessed the total and conjugated bilirubin level among the groups. The TPN group had significantly elevated total bilirubin (1.66 *vs.* 0.06 mg/dL; *p* = .040) and conjugated bilirubin (1.09 *vs.* 0.03 mg/dL; *p* = .029) (Figure 6(a)). Within the TPN group, TPH-HSI animals had a 4.84- and 13.7-fold increase in the total and conjugated bilirubin level, respectively compared to the TPN-LSI group (Figure 6(b,c)).

Within the TPN group, the TPN-LSI animals had a statistically significant reduction in total bile acids compared to TPN-HSI (8.27 *vs.* 60.4 mg/dL; *p* = .018) (Figure 6(d)). The serum bile acid level of TPN-LSI was not statistically different in comparison to the EN group (*p* = .741). Using our LPS discriminatory cut-off, EN animals with lower inflammation had a significant decrease in serum bile acids (5.15 *vs.* 10.5 mg/dL; *p* = .020) (Figure 6(e)).

The TPN animals had a significantly elevated triglyceride (44.0 *vs.* 27.2 mg/dL; *p* = .029) and VLDL (8.86 *vs.* 5.56 mg/dL; *p* = .033) and significantly decreased HDL (39.9 *vs.* 53.7 mg/dL; *p* = .014) (Figure S5). Within the TPN group, the TPN-HSI had a 1.40-, 1.61-, and 1.58-fold increase in the serum cholesterol, triglycerides, and VLDL, respectively *vs.* TPN-LSI but these did not reach statistical significance (Figure S5). While serum gamma-glutamyl transferase (GGT) was higher in TPN animals *vs.* EN, this did not reach statistical significance. No differences in ALT, AST, or alkaline phosphatase were noted between the TPN groups.

### 3.6. Markers for gut injury

**Figure 7.**
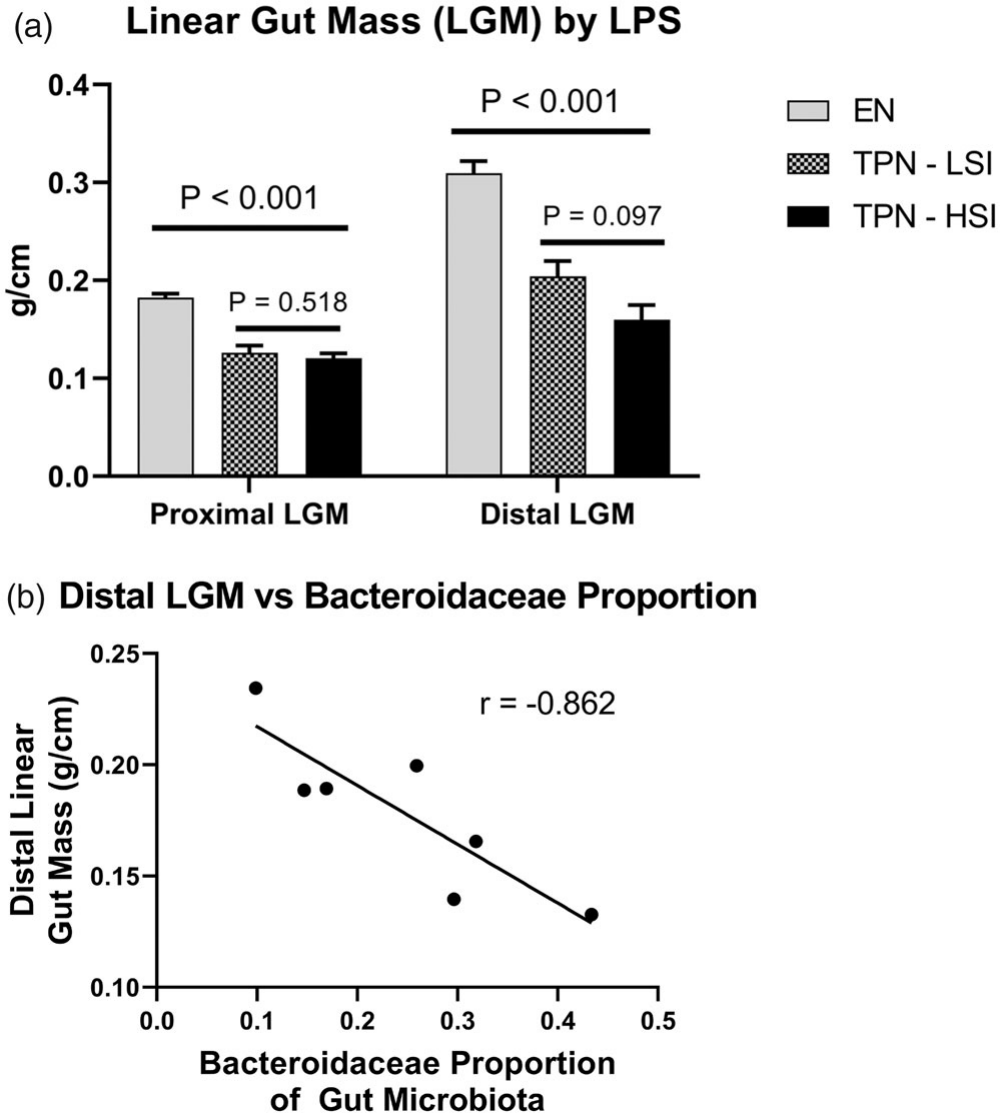
(A) Linear gut mass. Pigs on TPN had a significant decrease in the linear gut mass of the proximal gut and the distal gut compared to pigs fed enterally. No significant changes were seen in the linear gut mass between TPN-LSI and TPN-HSI. Pairwise *t*-test and ANOVA test were performed to determine the *p*-values. All tests were two-sided using a significance level of 0.05. (B) Distal Linear gut mass *vs.* Bacteroidaceae proportion. Among the TPN animals, the distal linear gut mass had a strong negative correlation with the proportion of Bacteroidaceae (*r* = −0.862).

Due to lack of intraluminal EN, TPN is known to drive gut atrophy, thus, to compare bowel growth among the groups we calculated the weight per centimetre of the distal and proximal small bowel segments to compute the linear gut mass (LGM) as previously published [3,6,17]. Compared to EN, animals on TPN had a significant decrease in the LGM of the proximal gut (0.183 *vs.* 0.123 g/cm; *p* < .001) and the distal gut (0.310 *vs.* 0.179 g/cm; *p* < .001). Between TPN-LSI and TPN-HSI, there were no significant changes in the proximal LGM (*p* = .518) or distal LGM (*p* = .097) (Figure 7(a)). Additionally, the distal LGM had a strong negative correlation with the level of Bacteroidaceae (*r* = −0.862) in TPN animals (Figure 7(b)).

## 4. Discussion

Using a novel ambulatory piglet TPN model, we noted gut microbial clonal shifts and systemic inflammation with TPN (Figure S6). High levels of inflammation had adverse effects on overall weight, gut growth as well as cholestasis. LPS is a component of the cell wall in Gram-negative bacteria and TPN promoted the expansion of many families of Gram-negative bacteria, such as *Bacteroidaceae*, *Fusobacteriaceae*, and *Campylobacteraceae* and a decrease of Gram-positive *Firmicutes* bacteria. Elevated portal serum levels of LPS were accompanied by significantly increased inflammatory cytokines IL-1b, IL-6, IL-10, IFN-g, and TNF-A in TPN animals. In animal cohorts with higher inflammation, the proportion of pathogenic Gram-negative bacteria families was more dominant. Pertinently, accompanying these microbial shifts, we observed that in TPN animals with higher systemic inflammation, serum surrogated of hepatic cholestasis was elevated.

Inflammation is a known contributor to cholestasis as seen in many conditions, such as sepsis. While the aetiology of inflammation-mediated cholestasis is likely multifactorial, altered bile acid transporter signalling drives cholestatic liver injury. In addition to animals on TPN, increased bile acid levels were seen in EN animals with higher inflammation. Indeed, rodent studies in different model systems have noted that LPS and/or the inflammatory cytokines IL-1β, IL-6, and TNF-A reduce the expression of bile acid transporters: bile salt export pump (BSEP), multidrug resistance-associated protein 2 (Mrp2) and sodium taurocholate co-transporting polypeptide (Ntcp) [25].

### 4.1. Decreases in firmicutes lead to LPS endotoxemia

The composition of gut microbiota is well known to differ among many disease states. For instance, stool samples from patients with Crohn’s disease demonstrate both a reduction in the abundance and diversity of *Firmicutes* compared to controls [26]. Similarly, stools in patients with type 2 diabetes show a reduction in the proportion of *Firmicutes* as well as an increased proportion of *Bacteroidetes*. The shift away from *Firmicutes* towards *Bacteroidetes* driven injury perhaps relates to the differential gut microbial metabolic products.

Another study in infant twins discordant for TPN-induced injury describes a more general shift towards Gram-negative bacterial growth in the TPN-treated patients [27]. The impact of these microbiota shifts on the host response is a developing area of research, and recent experiments have suggested that the impact of microbiota shifts may extend beyond nutritional uptake. For instance, certain Gram-negative species have been shown to impact the gut—Proteobacteria predominance in TPN has been associated with a pro-inflammatory state of IL-8 expression, and the presence of certain *Bacteroidetes* species has been linked to both inflammation and impairment of the gut mucosal barrier [28]. TPN administration is thought to cause a shift towards Gram-negative bacterial growth, which creates an inflammatory state through LPS release, leading to epithelial cell atrophy and a subsequent loss of epithelial barrier function [29].

Consistent with these findings, in our piglet model, we observed that TPN resulted in a loss of Gram-positive *Firmicutes* and the growth of many families of LPS releasing Gram-negative bacteria, such as *Bacteroidaceae*, *Fusobacteriaceae*, and *Campylobacteraceae*. As discussed, this dysregulation in the gut microbiota is associated with significant endotoxemia.

Additionally, in particular, among *Firmicutes, Ruminococcaceae* species are some of the primary producers of butyrate and have a pronounced reduction with TPN. Butyrate is a crucial energy source for the colonocytes that helps keep the epithelium intact. A loss of these SCFA-producing species combined with the increase in LPS containing Gram-negative bacteria contributed to the metabolic endotoxemia and inflammatory cytokine increase in animals on TPN.

### 4.2. Role of short-chain fatty acids

Short-chain fatty acids (SCFAs) are metabolites produced by microbial communities within the gut. These metabolic products include acetate, propionate, and butyrate, and result from the anaerobic fermentation of non-digestible carbohydrates by microbial communities. SCFAs, can directly activate intraluminal G-protein coupled receptors (GPCRs) and inhibit histone deacetylases, affecting a wide range of physiological processes contributing to health and disease. Additionally, SCFAs have a role in immune cell development as well as suppression of inflammation [30]. Reduced levels of SCFAs are often indicative of diseases including, but not limited to diabetes, obesity, autoimmune disorders, cancers, and various gastrointestinal disorders [31].

Butyrate is used primarily by the intestinal gut mucosal cells as the preferred energy source—serving up to 70% of enterocyte nutrition. It is generated from carbohydrates *via* the combination of two molecules of acetyl-CoA, forming acetoacetyl CoA, and then reduction to butyryl CoA, which forms butyrate *via* butyryl CoA:acetate CoA-transferase or *via* phosphotransbutyrylase and butyrate kinase. Butyrate-producing species belong to the two main families of human colonic *Firmicutes*, *Ruminococcaceae*, and *Lachnospiraceae* [30].

Propionate, on the other hand, is used in gluconeogenesis within the liver. It is mainly produced *via* the succinate pathway requiring vitamin B_12_ in *Bacteroidetes* and in the *Negativicutes* class of *Firmicutes*, *via* the propanediol pathway in *Lachnospiraceae*, and *via* acrylate pathways in *Negativicutes* and *Lachnospiraceae*. Propionate and butyrate may also be produced from amino acid fermentation in a small percentage of gut microbiota [30].

Acetate, a substrate used in producing butyrate, is found in the highest concentration in the blood. Butyrate and propionate both serve beneficial roles in maintaining overall health and are produced by discrete groups of the gut microbiome. In contrast, acetate is a fermentation product of most gut anaerobes, having the highest concentration within the lumen of the gut of any SCFA. These three also differ in their interactions with proteins and receptors within the host. These contrasting fates and interactions call for consideration of these SCFAs specific microbial origins as well as the possibility of variations in diet or gut functioning to disturb the relative concentrations and production rates [30].

The host diet affects the make-up and metabolism of gut microbiota. Dietary intake determines the capacity of the microbiome to produce metabolites, providing a link between the diet and different physiological states *via* microbiota composition. A diverse gut microbiome is influenced primarily by a diet rich in complex carbohydrates. Contrastingly, enduring high-fat and high-sucrose intake leads to the extinction of several taxa of the gut microbiota. Dietary fibres are the most prominent species fermented into SCFAs, but a small amount of SCFAs can be produced using protein fermentation as well [32].

In addition to their aforementioned roles, SCFAs have been shown to induce the assembly of tight junction proteins zonula occludens 1 (ZO-1) and occludin through an AMPK-dependent pathway in the gut. These tight junction proteins are critically regulated to manage molecular transport; in pathological settings, however, their assembly is dysregulated [33–35]. As a result, the endothelial tight junctions become more permeable, allowing for leakage. Indeed, increased permeability of the endothelial barrier has been implicated in several gastrointestinal and metabolic conditions, from inflammatory bowel disease to type II diabetes, and particularly NASH and other disorders of liver injury [33].

Critically in the setting of TPN, given the liver’s anatomical connection to the intestines *via* the portal vein, signalling molecules and inflammatory signals reach the liver at high levels [28,29]. One of these molecules is the bacterial-derived endotoxin LPS. While LPS is regularly circulating at low levels, in the setting of TPN, Gram-negative organisms are thought to have the clonal expansion, leading to increased release of LPS, which is then able to stimulate Kupffer cells to produce proinflammatory cytokines, such as IL-1β, IL-6, IL-8, and TFN-A. This creates a state of metabolic endotoxemia, contributing to characteristic TPN-induced injury [33,36–38].

Our further research focus is to use metagenomics data to assess the representation of genes encoding key SCFA metabolizing enzymes, to help correlate changes in gene abundance with specific bacterial phylum, families, and their subsequent lineage, as well as to further correlate genes based on clustering of other enzymatic protein sequences.

Another exciting area for further exploration is an analysis of microbial DNA from the rectum, colon, proximal and distal small bowel lumen, and wall scraping, to assess the region-specific impact of microbiota.

### 4.3. Systemic effects of TPN-induced inflammation

In our model, the prevalence of inflammatory mediators had adverse systemic effects. In both the control and TPN groups, as the level of LPS and inflammatory cytokines increased, the weight of the neonatal pigs on the day of the sac was decreased. For these neonatal pigs, the ability to gain weight is an indicator of the ability to thrive. Several studies have correlated TNF-A with hepatic inflammation and injury [39,40]. In the EN control group, TNF-A levels had the strongest correlation with the serum GGT levels, a marker of liver injury. In the TPN piglets, this trend was not as strong, but inflammatory cytokines had a positive correlation with GGT. In both EN and TPN animals, there was a statistically significant association between portal LPS levels and bile acid levels. In animals on TPN, serum Fibroblast Growth Factor 19 (FGF19) levels are decreased due to a lack of luminal Farnesoid X Receptor (FXR) activation [6,24]. FGF19 regulates CyP7A1, the rate-limiting step of bile acid synthesis, and is known to suppress CyP7A1. Thus, while LPS is known to suppress CyP7A1, the plausible explanation for the higher bile acid levels is due to the lack of the inhibition of CyP7A1 by FGF19 which is not offset by the LPS action.

Additionally, in TPN animals, we observed significant reductions in linear gut mass in the proximal and distal gut compared to control animals. Within the TPN group, the gut bacteria and inflammatory cytokines strongly correlated with gut mass. In particular, high levels of *Bacteroidaceae* species had a strong negative correlation with the linear gut mass in TPN animals [41]. Indeed, a lack of *Bacteroidaceae* and higher levels of *Firmicutes* have a gut protective effect. These findings suggest the critical role gut microbiota plays in liver injury and the state of metabolic endotoxemia complemented by the loss of protective SCFA-producing bacteria.

### 4.4. Limitations and future plans

While large animal studies like our piglet model recapitulate the human TPN state they are resource intensive. A limitation is the relatively small number of animals on TPN. Especially for subgroup analysis, a larger number of animals in the TPN group would have strengthened the data, which remains the focus of future work. Another limitation of the study is the lack of more functional gut microbiota data. The analyzed samples were stool, which may not be representative of the entire living microbiota in the gut. However, microbial metabolomics remains a key focus of future studies, including studies structured around inhibiting microbial colonies using targeted antibiotics as well as exploring outcomes of faecal microbial transfer from EN to those on TPN.

## 5. Conclusions

In summary, while TPN can cause severe liver injury, cholestasis, and gut atrophy, some subjects have a milder injury. Our study using a novel TPN piglet model showcases a novel link between gut bacteria, systemic inflammatory markers, and cholestasis. Pertinently, the most interesting finding in this study is the differential link between microbial clonal shifts and injury mechanisms—the higher the gut dysbiosis, the worse the systemic inflammation. Indeed, higher levels of *Firmicutes* species correlate with reduced systemic inflammation. Gram-negative bacteria families, such as *Bacteroidaceae*, *Fusobacteriaceae*, *Campylobacteraceae*, and *Burkholderiaceae* are associated with increased LPS and inflammatory cytokines.

## Supplementary Material

Supplemental Material

## Data Availability

Data generated or analyzed during this study are included in this article. Further enquires can be directed to the authors.

## Abbreviations

PN: parenteral nutrition
JV: jugular vein
DC: duodenal catheters
USDA: United States Department of Agriculture
CDCA: chenodeoxycholic acid
BSEP: bile salt export pump
Mrp2: multidrug resistance associated protein 2
Ntcp: sodium taurocholate co-transporting polypeptide
SCFA: short chain fatty acid
PNALD: parenteral associated liver disease
