# Supplementary material for: Lower systemic inflammation is associated with gut firmicutes dominance and reduced liver injury in a novel ambulatory model of parenteral nutrition
Source: Ann Med. 2022 Jun 15;54(1):1701–13. doi: 10.1080/07853890.2022.2081871 (PMC9225736; doi:10.1080/07853890.2022.2081871)
Supplement: Supplemental Material [file IANN_A_2081871_SM5142.pdf]

Figure S1

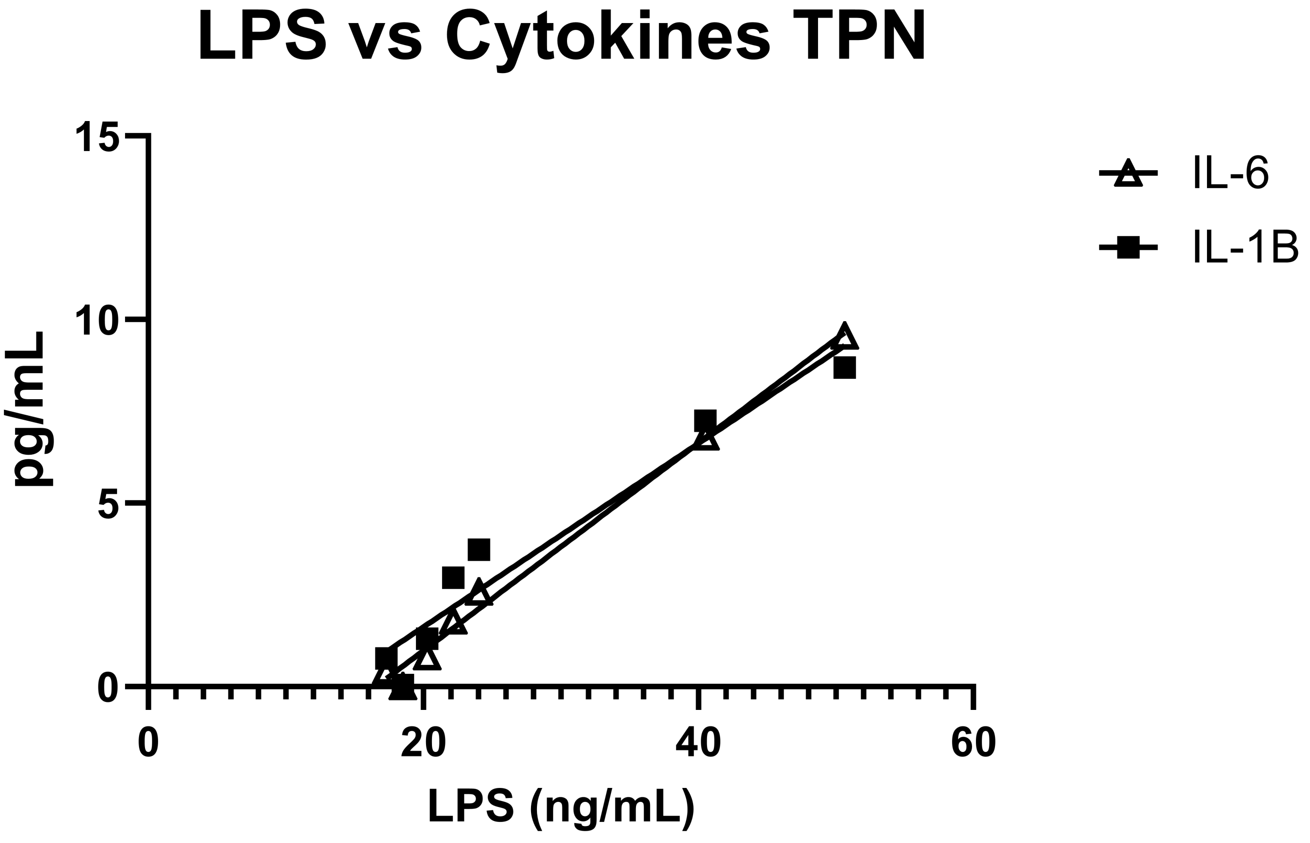

Figure S2

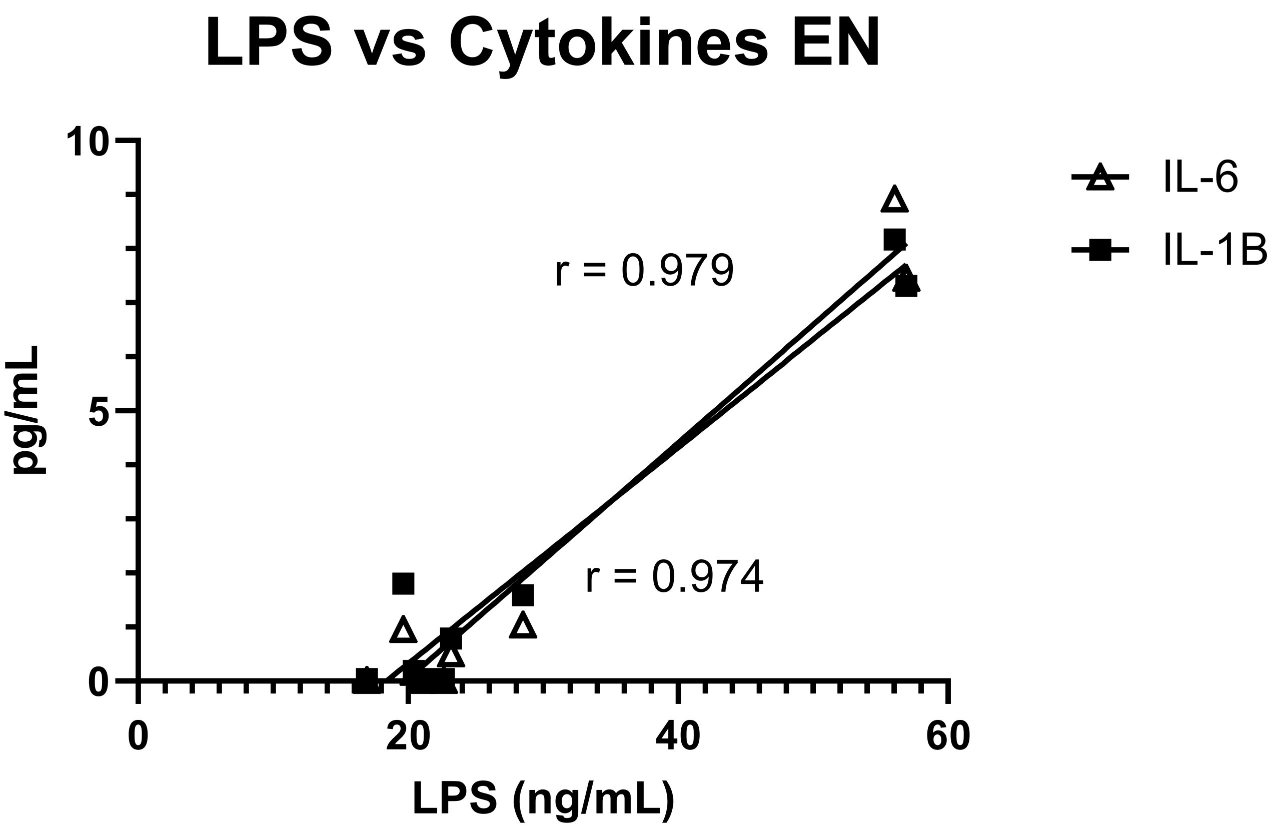

LPS vs Cytokines EN
10
5
0
pg/mL
0
20
40
60
LPS (ng/mL)
r = 0.979
r = 0.974
IL-6
IL-1B

Figure S3

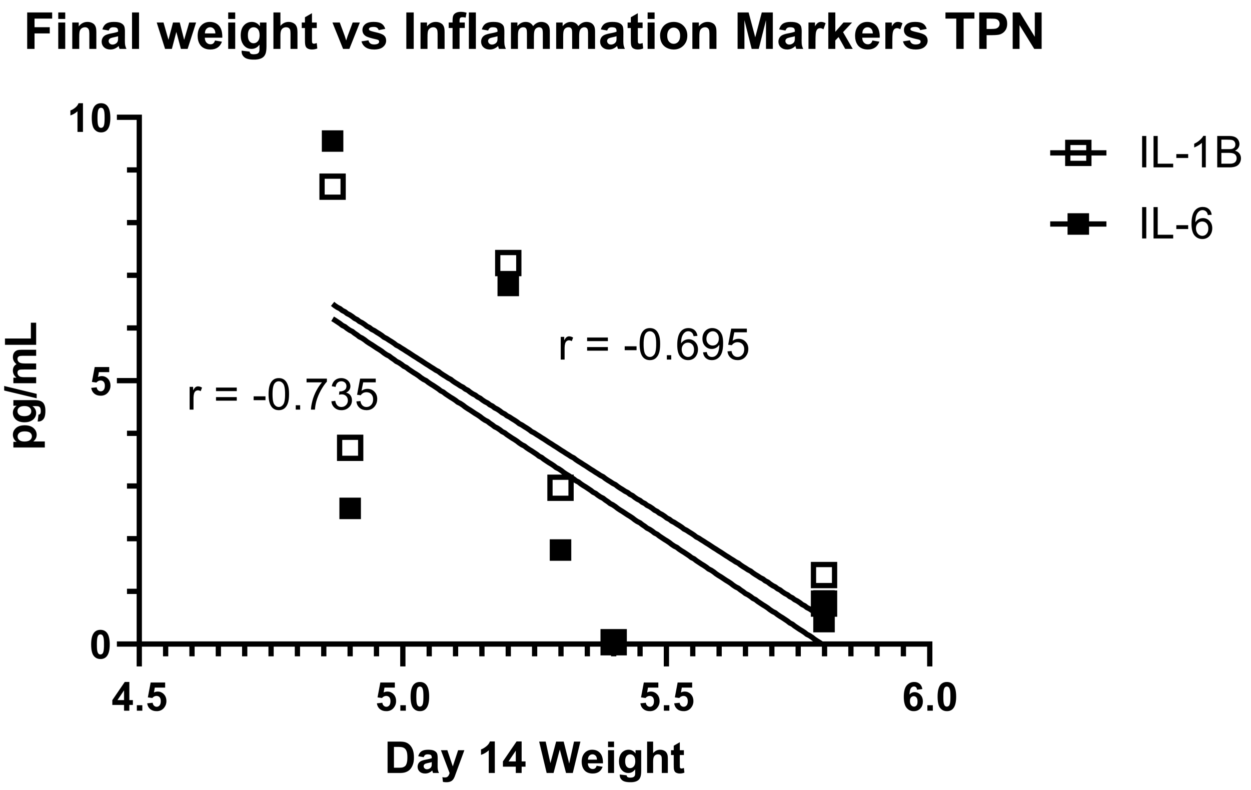

Final weight vs Inflammation Markers TPN
10
5
0
pg/mL
4.5
5.0
5.5
6.0
Day 14 Weight
r = -0.735
r = -0.695
IL-1B
IL-6

Figure S4

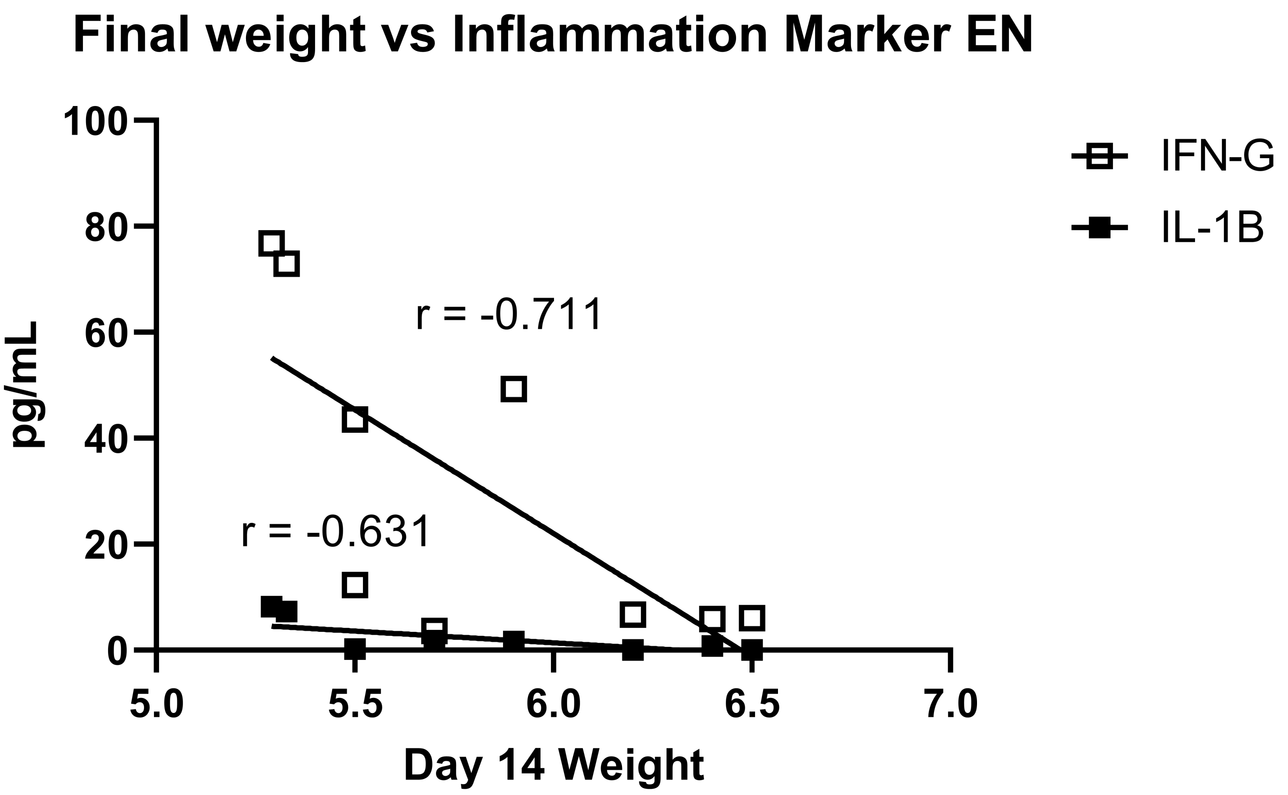
Final weight vs Inflammation Marker EN
pg/mL
100
80
60
40
20
0
5.0
5.5
6.0
6.5
7.0
Day 14 Weight
r = -0.711
r = -0.631
IFN-G
IL-1B

Figure S5

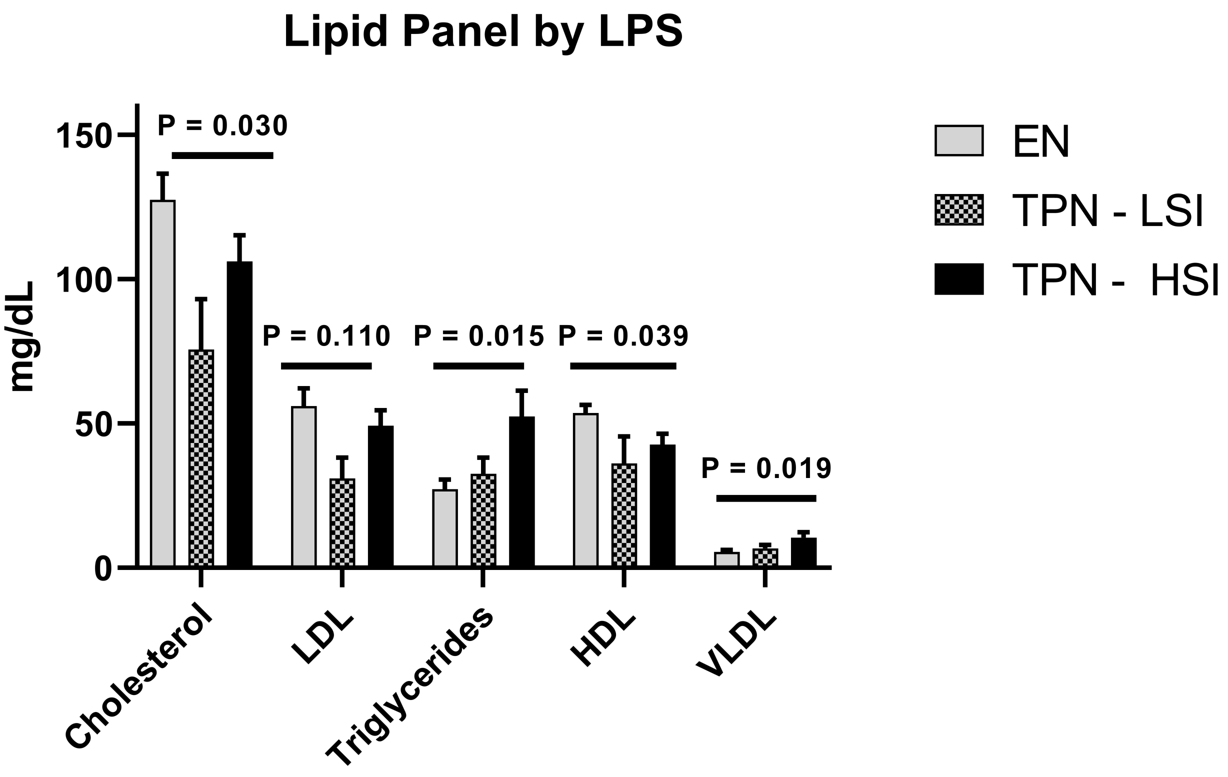
Lipid Panel by LPS
mg/dL
150
100
50
0
P = 0.030
P = 0.110
P = 0.015
P = 0.039
P = 0.019
Cholesterol
LDL
Triglycerides
HDL
VLDL
EN
TPN - LSI
TPN - HSI

Figure S6

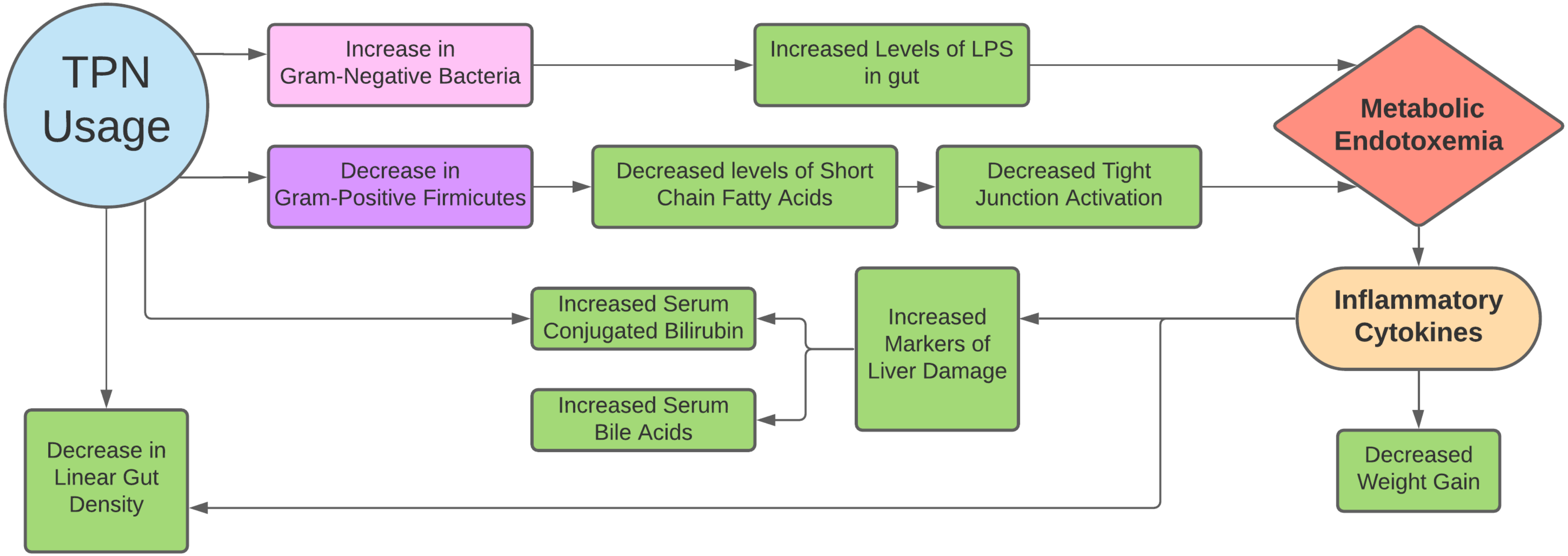

TPN Usage
Increase in Gram-Negative Bacteria
Increased Levels of LPS in gut
Metabolic Endotoxemia
Decrease in Gram-Positive Firmicutes
Decreased levels of Short Chain Fatty Acids
Decreased Tight Junction Activation
Increased Serum Conjugated Bilirubin
Increased Markers of Liver Damage
Inflammatory Cytokines
Increased Serum Bile Acids
Decrease in Linear Gut Density
Decreased Weight Gain
